# Supplementary figures and images for: Evaluating liver type fatty acid binding protein as a diagnostic and prognostic biomarker in metabolic dysfunction-associated steatotic liver disease in pediatric patients
Source: PLoS One. 2025 Sep 30;20(9):e0333581. doi: 10.1371/journal.pone.0333581 (PMC12483236; doi:10.1371/journal.pone.0333581)

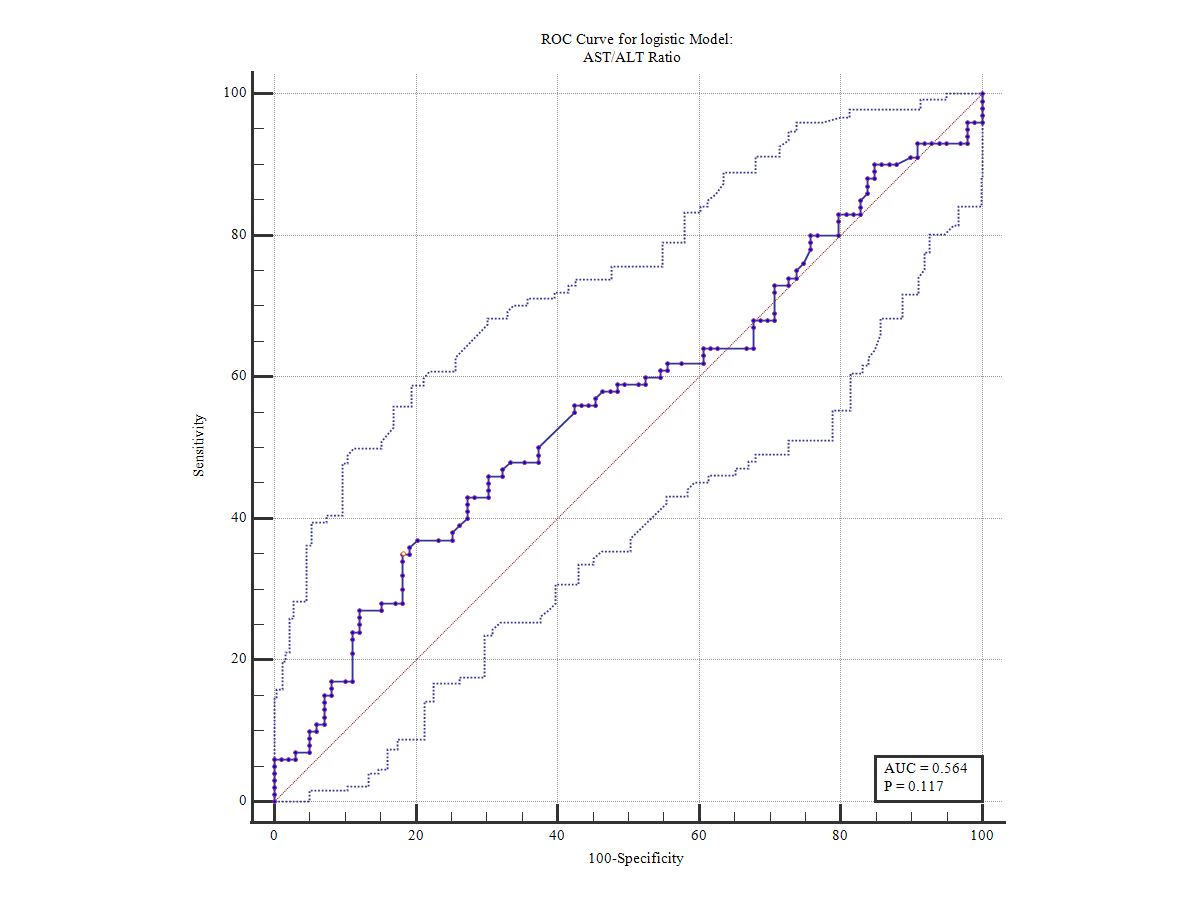

Supplement: S1 Fig — (TIF) [file pone.0333581.s001.tif]

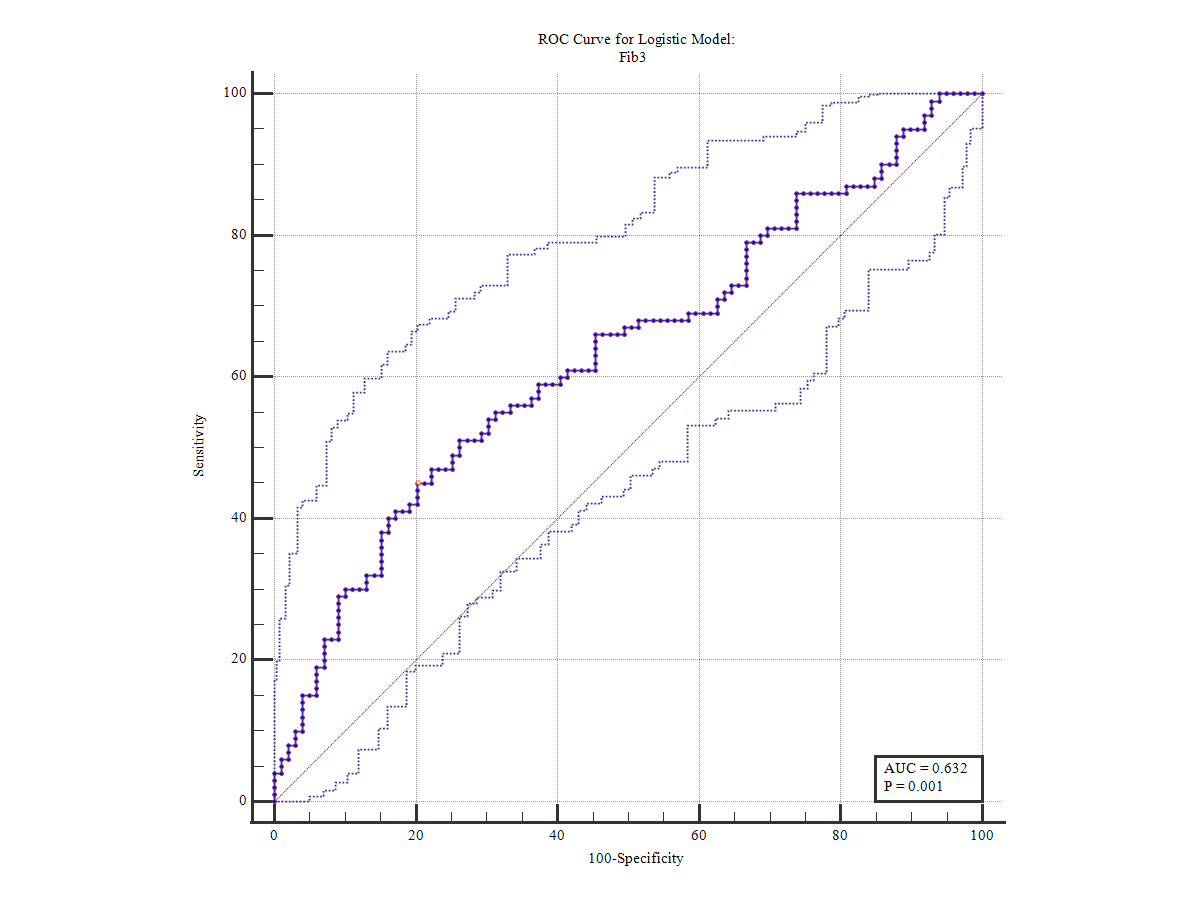

Supplement: S2 Fig — (TIF) [file pone.0333581.s002.tif]

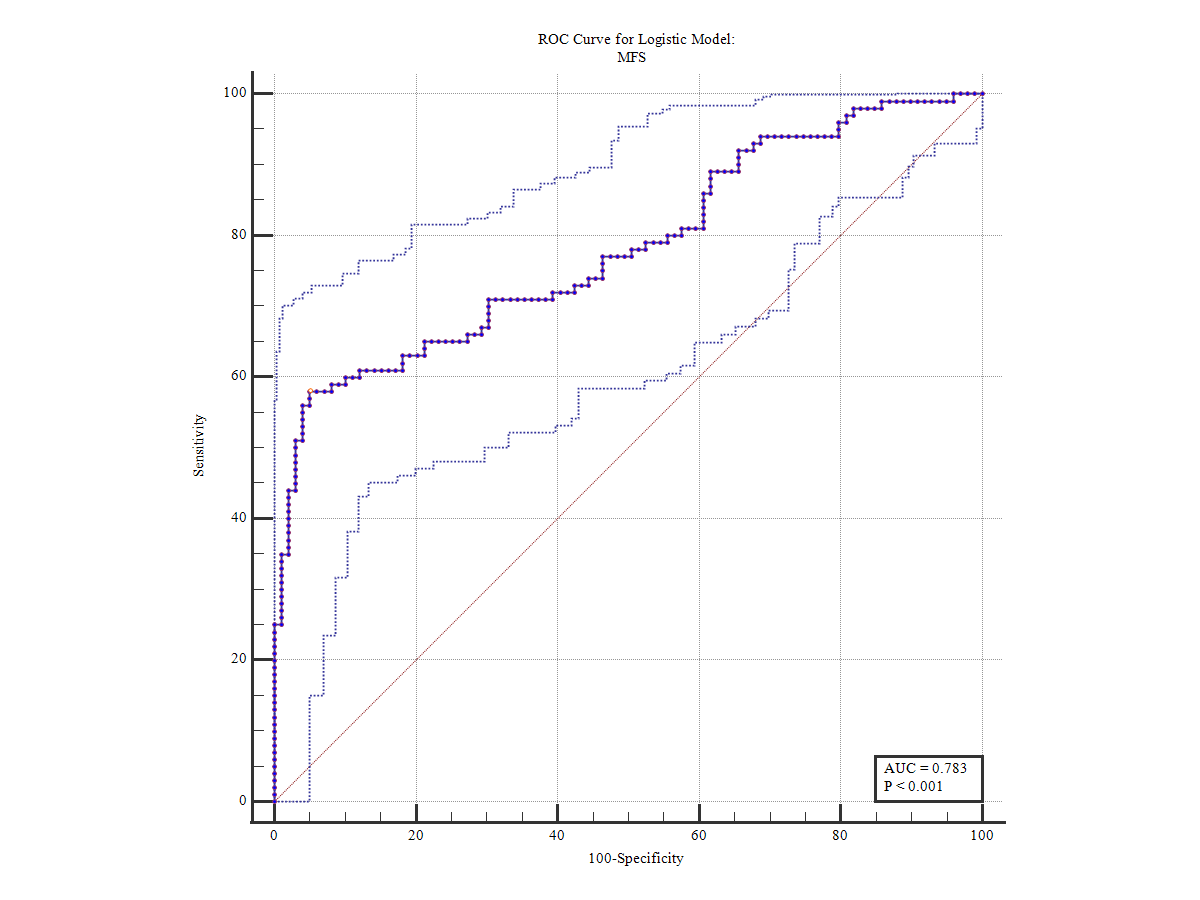

Supplement: S3 Fig — (TIF) [file pone.0333581.s003.tif]
