# Supplementary material for: Evaluating liver type fatty acid binding protein as a diagnostic and prognostic biomarker in metabolic dysfunction-associated steatotic liver disease in pediatric patients
Source: PLoS One. 2025 Sep 30;20(9):e0333581. doi: 10.1371/journal.pone.0333581 (PMC12483236; doi:10.1371/journal.pone.0333581)
Supplement: Attachment — Submitted filename: Point to Point Response.docx [file pone.0333581.s007.docx]

Rebuttal Letter

Marafi Jammaa Ahmed
[marafi.ahmed99@gmail.com](mailto:marafi.ahmed99@gmail.com)

To: **PLOS ONE** <[em@editorialmanager.com](mailto:em@editorialmanager.com)>
Subject: Rebuttal Letter for Manuscript Submission “Evaluating Liver Type Fatty Acid Binding Protein as a Diagnostic and Prognostic Biomarker in Metabolic dysfunction-associated steatotic liver disease in Pediatric Patients , PONE-D-24-57390”

Dear Esteemed Editors,

We sincerely appreciate the time and effort invested by the reviewers in evaluating our manuscript, Evaluating Liver Type Fatty Acid Binding Protein as a Diagnostic and Prognostic Biomarker in Metabolic dysfunction-associated steatotic liver disease in Pediatric Patients ". We are grateful for their insightful comments, which have helped improve the clarity, accuracy, and scientific rigor of our study.

Below, we provide a point-by-point response to each of the reviewers' comments, detailing the modifications made to the manuscript. All changes have been highlighted in the revised document for clarity.

**Point to Point Response**

| **Reviewer #1: Comments** | |
| --- | --- |
| 1. I would advise providing an epidemiologic data for children such as “with an estimation of 31.8% of all adults” instead of going with “Egypt has a large number of NAFLD pediatrics”. | The phrase *"Egypt has a large number of NAFLD pediatrics"* has been revised to incorporate specific epidemiologic data. Based on available studies, NAFLD prevalence in Egyptian schoolchildren is reported at 15.8%, while among healthy college students, it reaches 31.8%. However, epidemiologic data on pediatric NAFLD in Egypt remains scarce, highlighting the need for further research to investigate this aspect and provide a clearer understanding of disease prevalence and risk factors in this population. |
| 2. “NAFLD is anticipated to be one of the most common leading causes for liver transplantation” Does this information applies to children too? If liver transplantation is not a huge problem for children, I would advise going with different epidemiologic data | The original statement regarding NAFLD as a leading cause of liver transplantation was amended based on the recommendation. The revised version now focuses on disease progression in pediatric patients, emphasizing the risk of fibrosis and cirrhosis rather than liver transplantation, which is rare in children. This adjustment ensures the statement is more clinically relevant and aligns with pediatric NAFLD outcomes. |
| 3. I would revise this paragraph- “Liver function tests (LFTs) are needed for diagnosing and following up the liver status. Elevated aspartate transaminase (AST) and alanine transaminase (ALT), typically one to four times the upper limits of normal, are the most frequently observed abnormal laboratory test results. However, Patients with nonalcoholic fatty liver disease, or mild steatosis may have normal or slightly elevated transaminase levels”. Instead, please provide more info if liver enzymes by themselves are good enough to detect NAFLD, or we need more sophisticated laboratory screening parameters. For example, FIB-4 or other currently available biomarkers can be useful in children, or not? | The paragraph discussing liver function tests (LFTs) for diagnosing and monitoring liver status has been revised to include additional information on the limitations of ALT and AST in detecting NAFLD. The updated version clarifies that while ALT and AST are commonly used markers, they may remain normal in some cases, particularly in mild steatosis. The revision also introduces more sophisticated non-invasive biomarkers, such as FIB-4, NAFLD Fibrosis Score, and Enhanced Liver Fibrosis (ELF) test, discussing their potential utility in pediatric patients. This ensures the text provides a more comprehensive view of diagnostic approaches beyond routine LFTs. |
| 4. I am wondering if the change in terminology of NAFLD to MASLD applies to children as well, or not. If yes, can we update the terminology here? | Based on the updated guidelines and recent expert perspectives, the terminology change from NAFLD to MASLD applies to pediatric patients as well. The manuscript has been revised accordingly to reflect this updated nomenclature, ensuring consistency with the latest clinical recommendations in pediatric hepatology. The changes also align with the broader classification of Steatotic Liver Disease (SLD), which now includes MASLD and MASH (previously NASH). |
| 5. “stages from NAFLD to hepatocellular carcinoma” instead of indicating HCC, maybe it would be better to indicate from NAFLD to cirrhosis, or even with complications such as HCC! | The phrase “stages from NAFLD to hepatocellular carcinoma (HCC)” has been revised for better accuracy. Since HCC is rare in pediatric patients, the updated wording now describes the progression from MASLD (formerly NAFLD) to cirrhosis, with potential complications, including HCC. This revision better reflects the natural history of the disease in children, where the primary concern is the development of fibrosis and cirrhosis, which can lead to complications later in life. |
| 6. I would suggest revising the aim: “The aim of this study was to investigate the potential association between the serum levels of L FABP and the liver functions. Moreover, to assess the use of L-FABP serum concentration as a prognostic and diagnostic biomarker in pediatric NAFLD patients to monitor the extent of fibrosis”. Based on your introduction, you are trying to detect NAFLD with advanced fibrosis, right? However, “The inclusion criteria were: Asymptomatic patients or with a history of mild or moderate liver disease” you included only mild to moderate patients. So, your biomarker based on the studies are beneficial to detect overall NAFLD, or NAFLD with advanced disease. If advanced disease, you only discussed in your introduction how it is beneficial to catch severe presentations. So, you need to revise the introduction as well. | Thank you for your valuable feedback. We acknowledge the need for clarification regarding the study aim and inclusion criteria. By "mild to moderate" in the inclusion section, we intended to encompass all stages of liver fibrosis except for cirrhosis. Consequently, some patients with advanced fibrosis were indeed enrolled. To accurately reflect our study's actual scope and enrollment, we have now amended the description of the inclusion criteria to explicitly state this intent. Additionally, we have updated the exclusion criteria to explicitly mention cirrhosis, ensuring consistency with our actual study population.  In light of this, we have revised the introduction to better align with our study's focus—not just on detecting NAFLD in general but also on assessing L-FABP as a biomarker for identifying varying degrees of fibrosis, including advanced fibrosis before cirrhosis develops. This clarification ensures that our study's aim and design are clearly communicated and accurately reflect the population we investigated.  We appreciate your insightful comments, which have helped refine the clarity and precision of our manuscript. |
| 7. What’d you mean you have excluded the patients with “elevated transaminases”? Then, how come you aimed to investigate the relation with elevated trans aminases and your biomarker relation? | The manuscript has been clarified to specify that only elevated transaminases indicating an acute liver disease were excluded |
| 8. If those are mild to moderate NAFLD patients, why did you measure AFP values? | AFP was measured to explore whether subtle elevations occur in pediatric NAFLD, even in the absence of malignancy. However, its primary role remains in detecting hepatocellular carcinoma, which is rare in children. (There was a significant difference between control and test groups, ***P-value*** <0.0001) |
| **Reviewer # 2: Comments** | |
| (1) What is the rationale for using the term "Non-Alcoholic Fatty Liver Disease", when the international Hepatology community has reached consensus to move to "Metabolic Dysfunction-Associated Steatotic Liver Disease"? | The terminology “Non-Alcoholic Fatty Liver Disease (NAFLD)” was used in the study because, at the time the research was initiated, the transition to “Metabolic Dysfunction-Associated Steatotic Liver Disease (MASLD)” was still under debate within the international hepatology community.  Since then, a global consensus has been reached in favor of MASLD as the preferred term. Accordingly, the manuscript has been updated to reflect this terminology change where applicable, ensuring alignment with current hepatology guidelines while acknowledging that the study was conducted during the transitional period in nomenclature. |
| (2) It is not clear how "NAFLD patients" and "controls" were recruited or have been assigned to their respective groups. What were the inclusion criteria for each? Is the "patient" group defined on biopsy, or on non-invasive assessment? If the latter, it does not take anything away from the findings but should be clarified. How about controls? Have they had any assessment to exclude steatosis, which is, after all, a silent condition? | The recruitment process is detailed in the Methods section. MASLD patients were selected based on predefined staging criteria. Inclusion criteria included asymptomatic or mild/moderate liver disease cases, normal INR, age >2.5 to <18 years, and confirmed MASLD diagnosis based on predefined staging. Exclusion criteria included fulminant/acute liver failure, viral hepatitis (HCV, HBV), hepatocellular carcinoma, or refusal of consent. The control group consisted of healthy children with no clinical signs of MASLD. We confirm that controls underwent appropriate assessments, including ultrasound imaging, to exclude hepatic steatosis, given its asymptomatic nature. The extent of fibrosis and the level of steatosis was based on liver brightness in addition to a transient elastography fibroscan (Abdominal examination section) The recruitment process was further clarified and amended in the manuscript (lines 163-167) |
| (3) What criteria were used to define "NAFLD stages"? This should be clarified in the methods section. | We utilized transient elastography (FibroScan®) to determine MASLD stages (F0-F3). This classification is detailed in the Methods section and aligns with established non-invasive fibrosis staging methods. Amended in the manuscript (lines 196-200) |
| (4) The authors put patients into 4 categories based on ultrasonographic assessment. It is not clear what the rationale for these headings is - these headings in turn form the basis for Figure 1. It is not clear why "bright hepatomegaly" and "fatty liver" are listed as different groups. Similarly, does the term "coarse liver" imply that these patients have cirrhosis? This seems unlikely given that the proportion with "coarse liver" is very high, and it does not correlate with the separately reported "NAFLD stages" - although it is not clear how those stages were defined. | Thank you for your insightful comments. We appreciate the opportunity to clarify the rationale behind the ultrasonographic categories used in our study. These four categories—bright hepatomegaly, coarse liver, fatty liver, and splenomegaly—were defined based on differences in hepatic echotexture, as detailed in the manuscript (Lines 235-237).   - Bright hepatomegaly refers to increased echogenicity combined with hepatomegaly. - Fatty liver denotes uniform hepatic steatosis while maintaining a normal liver size. - Coarse liver represents advanced fibrosis but does not indicate cirrhosis. - Splenomegaly is included due to its association with liver disease progression.   To avoid any ambiguity, we have clarified these distinctions in the methodology section (Lines 196-200).  Importantly, we acknowledge that ultrasonography has limitations in precisely quantifying fibrosis compared to Fibroscan. Therefore, while ultrasonographic patterns were categorized based on echotexture, the degree of fibrosis in our study participants was primarily determined by Fibroscan results. The ultrasonographic findings were considered complementary but not definitive for fibrosis staging.  We have ensured that these clarifications are reflected in the revised manuscript to strengthen the coherence between the ultrasonographic classifications and fibrosis staging. We appreciate your feedback, which has helped refine the clarity and precision of our methodology. |
| (5) Lines 87 - 88 need revising. Younnossi et al, recognised as an authority in the field of MASLD epidemiology, put the global prevalence of MASLD at 30%. The references used refer to global NAFLD and paediatric NAFLD, and it is not clear what exactly is being referred to. I would urge further clarity here. | We acknowledge the inconsistency in prevalence estimates. Younossi et al. estimate the global prevalence of MASLD at 30%. We revised the manuscript to clearly differentiate between general MASLD prevalence and pediatric-specific estimates (lines 99-104). |
| (6) Line 119 - The aim of the study is to look at the association between L-FABP and "liver functions". It is not clear what the term "liver functions" refers to. If it refers to transaminases, then it should be clarified, although the authors argue later in the manuscript that transaminases are a poor reflection of liver function per se. Same comment for the phrase "liver function biomarkers" in line 173. | We replaced "liver functions" with a more precise term "hepatic biochemical markers" ensuring clarity regarding which parameters (ALT, AST, albumin, etc.) were evaluated. |
| (7) Line 149 - Could the authors clarify whether these were fasting blood samples or otherwise? It has implications for interpretation of parameters like triglycerides and glucose concentrations. | Yes, blood samples were collected under fasting conditions. We explicitly stated this in the Methods section, as fasting status affects parameters such as triglycerides and glucose levels (now line 177). |
| (8) Line 168 - Minor point - Are the authors sure that Fibroscan is a US brand (rather than French)? | We acknowledge the oversight regarding FibroScan's origin. It is a French brand developed by Echosens. We corrected this in the manuscript. |
| (9) Table 1 - I am not convinced there is a statistical difference in BMI between NAFLD cases and controls. Could the authors clarify the stats? They should also include standard deviation scores (sds) for BMI, or centiles, as is standard for reporting paediatric parameters. The row labelled "Sonography" - unclear what was calculated here | We appreciate the reviewer’s careful examination of Table 1 and for highlighting the statistical difference in BMI between NAFLD cases and controls. We have rechecked our analysis to ensure accuracy and there is a typographical error in the NAFLD Gp BMI. It was just a typo error in the table. The true value for NAFLD gp was (33.69 ± 4.57) not 23.69  This raw was amended to clarify that the statistical testing was performed between L-FABP level and the ultrasonographic categories of patients. |
| (10) Lines 263-267 - Here the authors discuss the utility of ALT in diagnosing and monitoring MASLD and refer to data from the adult population. Is this similarly true for the paediatric population? I think it is worth being more specific here, as this is the group of interest, and ALT in particular seems to vary across the life course. | We appreciate your insightful comments regarding the utility of alanine aminotransferase (ALT) in diagnosing and monitoring Metabolic Dysfunction-Associated Steatotic Liver Disease (MASLD), particularly in the pediatric population.  In light of these considerations, we proposed the following revisions to the manuscript to address the variability of ALT levels and its implications for diagnosing and monitoring MASLD in the pediatric population:  *Revised Text:*  "ALT levels vary significantly across the lifespan, influenced by factors such as age, sex, and pubertal status. In children and adolescents, ALT levels tend to rise during puberty, with boys exhibiting higher levels than girls. However, relying solely on ALT levels for diagnosing MASLD in the pediatric population has limitations, as a notable proportion of children with biopsy-confirmed MASLD may present with normal ALT levels. Therefore, while ALT can serve as an initial screening tool, particularly in children over 10 years of age with elevated BMI, it should be complemented with other diagnostic modalities. These may include imaging studies, such as ultrasound or magnetic resonance elastography, and the assessment of additional biomarkers to enhance diagnostic accuracy and effectively monitor disease progression in pediatric MASLD." (lines 310-318) |
| (11) Line 301 - Would the authors consider specifiying that L-FABP has "potential utility as a biomarker for the diagnosis of PAEDATRIC NAFLD"? | The manuscript has been updated to explicitly state that L-FABP has "potential utility as a biomarker for the diagnosis of PAEDATRIC MASLD" |
