# Supplementary material for: Evaluating liver type fatty acid binding protein as a diagnostic and prognostic biomarker in metabolic dysfunction-associated steatotic liver disease in pediatric patients
Source: PLoS One. 2025 Sep 30;20(9):e0333581. doi: 10.1371/journal.pone.0333581 (PMC12483236; doi:10.1371/journal.pone.0333581)
Supplement: Attachment — Submitted filename: Rebuttal Letter.docx [file pone.0333581.s008.docx]

Rebuttal Letter

Point to point response (Reviewer 5)

| Comment | Response |
| --- | --- |
| 1. The authors state that “liver biopsy is the gold standard in the diagnosis of MASLD”. However, the diagnosis of fatty liver in this study was made using abdominal ultrasound. At least, references should be provided on the utility of abdominal ultrasound and FibroScan^®^ in the diagnosis of MASLD in children. | Thank you for your insightful observation. We agree that liver biopsy remains the gold standard for diagnosing MASLD; however, due to its invasive nature and limited feasibility in pediatric populations, non-invasive modalities are now commonly used in clinical and research settings.  In our study, abdominal ultrasonography was performed as part of the routine imaging assessment to evaluate general liver morphology and identify overt hepatic steatosis. The diagnosis and staging of MASLD were based on transient elastography (FibroScan^®^ Compact 530),  To address your concern, we have clarified this distinction in the manuscript and added appropriate references supporting the clinical utility of FibroScan® in the evaluation of MASLD in pediatric populations (References 23–25). |
| 2. The definitions of “fatty liver” and “MASLD” were not shown. | Thank you for pointing that out. The definitions of “fatty liver” and “MASLD” were previously missing and have now been added at **line 90** for clarity and completeness. |
| 3. The bright hepatomegaly, coarse liver, fatty liver, and splenomegaly, which are indicated in Figure 1, need to be explained in the method. | We appreciate your suggestion and have revised the Methods section accordingly to include clear definitions of the ultrasound findings mentioned in Figure 1. Specifically, we now explain the terms “bright hepatomegaly,” “coarse liver,” “fatty liver,” and “splenomegaly” as part of the routine sonographic evaluation. **(line 202)** |
| 4. Table 2. Correlation coefficients, not just p-values, should be shown. | Pearson’s partial correlation coefficient is now added to the table (for univariable analysis while adjusting for group effect). |
| 5. To compare the ROC of L-FABP for diagnosis MASLD and that of BMI, fatty liver index, AST/ALT or ALT etc. | Thank you for this important comment. We acknowledge the potential interest in comparing L-FABP to other non-invasive markers such as BMI, fatty liver index (FLI), AST/ALT ratio, and ALT alone. However, as discussed in the subsequent comment, the utility and diagnostic validity of these parameters in pediatric populations remain limited and not well-established.  For example, the Fatty Liver Index was developed and validated in adults and has not been appropriately adapted or validated for use in children. Similarly, indices like AST/ALT ratio and isolated ALT measurements lack sufficient sensitivity and specificity for diagnosing MASLD or staging fibrosis in the pediatric setting. Given these limitations, we chose not to include these comparisons in our ROC analysis to avoid drawing potentially misleading conclusions. |
| 6. To compare the utility of L-FABP for diagnosis F2 or F3 stage and that of Fib-4 index, Fib-3 index, MASLD fibrosis score, or AST/ALT, etc. | Thank you for your insightful comment regarding the use of non-invasive fibrosis scores such as FIB-4, APRI, and NFS in the evaluation of MASLD. While these scoring systems are widely used and validated in adult populations, their utility in children remains limited and lacks sufficient validation.  The FIB-4 index—although well-established in adults—has reduced reliability in certain populations, particularly in individuals under 35 years of age, where it tends to underperform. This is especially relevant in pediatric cohorts, where age-related physiological differences affect the accuracy of these indices. Additionally, FIB-4’s diagnostic value is limited in the intermediate range and may result in false positives in populations with a lower prevalence of advanced fibrosis, further limiting its clinical applicability in younger age groups.  Given these constraints, and in alignment with current literature, we opted not to rely on FIB-4 or similar adult-derived scores in our pediatric MASLD study. <https://www.journal-of-hepatology.eu/article/S0168-8278(24)00329-5/fulltext>  <https://journals.lww.com/ajg/abstract/2017/05000/age_as_a_confounding_factor_for_the_accurate.21.aspx>  We appreciate your thoughtful feedback and trust this addresses your concern. |
