# Supplementary material for: Evaluating liver type fatty acid binding protein as a diagnostic and prognostic biomarker in metabolic dysfunction-associated steatotic liver disease in pediatric patients
Source: PLoS One. 2025 Sep 30;20(9):e0333581. doi: 10.1371/journal.pone.0333581 (PMC12483236; doi:10.1371/journal.pone.0333581)
Supplement: Attachment — Submitted filename: Response to Reviewers 14 JUNE 2025.docx [file pone.0333581.s009.docx]

**Response to Reviewers**

Point to point response

Dear Respected Editor

Good day

Thank you for the constructive comments, hereby were our responses.

**Comment**

**Reviewer's Responses to Questions from Comments to the Author**

**1.** If the authors have adequately addressed your comments raised in a previous round of review and you feel that this manuscript is now acceptable for publication, you may indicate that here to bypass the “Comments to the Author” section, enter your conflict of interest statement in the “Confidential to Editor” section, and submit your "Accept" recommendation.

Reviewer #5: All comments have been addressed.

**Response**

**Thanks**

**2. Is the manuscript technically sound, and do the data support the conclusions?**

The manuscript must describe a technically sound piece of scientific research with data that supports the conclusions. Experiments must have been conducted rigorously, with appropriate controls, replication, and sample sizes. The conclusions must be drawn appropriately based on the data presented.

Reviewer #5: Partly

**Response**

We assume this assessment is primarily based on the concern raised in point 6. In response, we have addressed this point thoroughly in our reply to comment 6. We hope that this added analysis satisfactorily addresses the reviewer’s concern and affirms the methodological rigor and data-supported conclusions of the study.

**3. Has the statistical analysis been performed appropriately and rigorously?**

Reviewer #5: Yes

**Response**

**Thanks**

**4. Have the authors made all data underlying the findings in their manuscript fully available?**

The [PLOS Data policy](http://track.editorialmanager.com/CL0/http:%2F%2Fwww.plosone.org%2Fstatic%2Fpolicies.action%23sharing/1/010f01972320f678-d57bfad4-4b12-4d01-af0a-6a49970a9245-000000/tt5DZGagXB_unsexjpuFpN3h-FwxOTdFuClS9Qe1E8A=213) requires authors to make all data underlying the findings described in their manuscript fully available without restriction, with rare exception (please refer to the Data Availability Statement in the manuscript PDF file). The data should be provided as part of the manuscript or its supporting information, or deposited to a public repository. For example, in addition to summary statistics, the data points behind means, medians and variance measures should be available. If there are restrictions on publicly sharing data—e.g. participant privacy or use of data from a third party—those must be specified.

Reviewer #5: Yes

**Response**

**Thanks**

5. Is the manuscript presented in an intelligible fashion and written in standard English?

PLOS ONE does not copyedit accepted manuscripts, so the language in submitted articles must be clear, correct, and unambiguous. Any typographical or grammatical errors should be corrected at revision, so please note any specific errors here.

Reviewer #5: Yes

**Response**

**Thanks**

6. Review Comments to the Author

Please use the space provided to explain your answers to the questions above. You may also include additional comments for the author, including concerns about dual publication, research ethics, or publication ethics. (Please upload your review as an attachment if it exceeds 20,000 characters)

Reviewer #5: This reviewer understands that BMI, fatty liver index, AST/ALT or ALT, Fib-4 index, Fib-3 index, and MASLD fibrosis score are useful in adults, but their usefulness in children has not been clearly established. The purpose of this study is to show the utility of L-FABP; thus, the authors should be compared these markers with L-FABP to clarify this point.

**Comment**

**Response**

We thank the reviewer for highlighting the importance of ensuring scientific rigor and drawing conclusions supported by robust data. In response, we would like to clarify that non-invasive fibrosis scores, including AST/ALT, Fib-4 index, Fib-3 index, and MAFLD fibrosis score, were indeed calculated and analyzed in our study. These scores were used to provide a comparative context for evaluating the diagnostic performance of L-FABP in detecting liver fibrosis among the pediatric MASLD population. Despite the fact that these indices were originally developed and validated in adult populations, we performed ROC curve analyses comparing them to L-FABP. This was done to assess whether L-FABP offers any additional diagnostic utility or improved performance in a pediatric setting. The results, which are now included in the revised manuscript [lines 296-302, 403-416, 422-424], show that L-FABP demonstrates superior diagnostic accuracy compared to the aforementioned scores in identifying children with stage of fibrosis. We believe that including this comparative analysis strengthens the validity of our conclusions and demonstrates that our findings are drawn from technically sound and appropriately controlled data.

7. PLOS authors have the option to publish the peer review history of their article ([what does this mean?](https://track.editorialmanager.com/CL0/https:%2F%2Fjournals.plos.org%2Fplosone%2Fs%2Feditorial-and-peer-review-process%23loc-peer-review-history/1/010f01972320f678-d57bfad4-4b12-4d01-af0a-6a49970a9245-000000/o1TMkISY1O6o67AMdsCFsBSBaFXQa3wp25GySrM5ZJU=213)). If published, this will include your full peer review and any attached files.

If you choose “no”, your identity will remain anonymous but your review may still be made public.

**YES.**
