# Supplementary material for: Evaluating liver type fatty acid binding protein as a diagnostic and prognostic biomarker in metabolic dysfunction-associated steatotic liver disease in pediatric patients
Source: PLoS One. 2025 Sep 30;20(9):e0333581. doi: 10.1371/journal.pone.0333581 (PMC12483236; doi:10.1371/journal.pone.0333581)
Supplement: Attachment — Submitted filename: Responses to Reviewers.docx [file pone.0333581.s010.docx]

**Response to Reviewers**

Point to point response

Dear Respected Editor

Good day

Thank you for the constructive comments, hereby were our responses.

**Comment**

**Reviewer's Responses to Questions from Comments to the Author**

**1.** If the authors have adequately addressed your comments raised in a previous round of review and you feel that this manuscript is now acceptable for publication, you may indicate that here to bypass the “Comments to the Author” section, enter your conflict of interest statement in the “Confidential to Editor” section, and submit your "Accept" recommendation.

Reviewer #6: (No Response)

**2. Is the manuscript technically sound, and do the data support the conclusions?**

The manuscript must describe a technically sound piece of scientific research with data that supports the conclusions. Experiments must have been conducted rigorously, with appropriate controls, replication, and sample sizes. The conclusions must be drawn appropriately based on the data presented.

Reviewer #5: Partly

**Response**

We assume this assessment is primarily based on the concern raised in points 3 and 4. In response, we have addressed this point thoroughly in our reply to comments 3 and 4. We hope that this added analysis satisfactorily addresses the reviewer’s concern and affirms the methodological rigor and data-supported conclusions of the study.

**3. Has the statistical analysis been performed appropriately and rigorously?**

Reviewer #6: No

**Response**

We assume this assessment is primarily based on the concern raised in point 6. In response, we have addressed this point thoroughly in reply to comment 6. We hope that this added analysis satisfactorily addresses the reply and affirms the statistical rigor and appropriateness.

**4. Have the authors made all data underlying the findings in their manuscript fully available?**

The [PLOS Data policy](http://track.editorialmanager.com/CL0/http:%2F%2Fwww.plosone.org%2Fstatic%2Fpolicies.action%23sharing/1/010f01972320f678-d57bfad4-4b12-4d01-af0a-6a49970a9245-000000/tt5DZGagXB_unsexjpuFpN3h-FwxOTdFuClS9Qe1E8A=213) requires authors to make all data underlying the findings described in their manuscript fully available without restriction, with rare exception (please refer to the Data Availability Statement in the manuscript PDF file). The data should be provided as part of the manuscript or its supporting information or deposited to a public repository. For example, in addition to summary statistics, the data points behind means, medians and variance measures should be available. If there are restrictions on publicly sharing data—e.g. participant privacy or use of data from a third party—those must be specified.

Reviewer #6: No

**Response**

We confirm that all raw data underlying the findings of this study have been provided as a supplementary file accompanying the manuscript. Additionally, a Data Availability Statement has been added to the manuscript, clarifying that all relevant data are fully available without restriction and can be accessed in the supplementary material.

**5. Is the manuscript presented in an intelligible fashion and written in standard English?**

PLOS ONE does not copyedit accepted manuscripts, so the language in submitted articles must be clear, correct, and unambiguous. Any typographical or grammatical errors should be corrected at revision, so please note any specific errors here.

Reviewer #6: Yes

**Response**

**Thanks**

6. Review Comments to the Author

Please use the space provided to explain your answers to the questions above. You may also include additional comments for the author, including concerns about dual publication, research ethics, or publication ethics. (Please upload your review as an attachment if it exceeds 20,000 characters)

Reviewer #6: The aim of this study is intriguing. However, this Reviewer has several major issues that should be addressed.

**Response to MAJOR REVISIONS**

**1. What about on-going pharmacological treatment? In discussion you mentioned UDCA as possible modifier of ALT, but no information are given about treatment in study population.
Response**

We appreciate the reviewer’s valuable comment regarding pharmacological treatment. We have now clarified in the **Patients and Study Design** section that all enrolled patients received treatment protocols as prescribed by their managing physicians, with no interference from the research team as this was an observational non-interventional study. Importantly, none of the participants were receiving ursodeoxycholic acid (UDCA) or other hepatoprotective agents that could influence ALT or L-FABP levels.

Additionally, we would like to emphasize that by the time of the study, there were currently no pharmacological agents approved specifically for the treatment of MASLD (previously NAFLD) in the pediatric population. The cornerstone of management remains lifestyle modification, which includes gradual weight reduction (if needed), adherence to a balanced diet low in added sugars and saturated fats, and engagement in regular physical activity. These interventions were encouraged as part of routine clinical care for the patients but were not controlled or modified by the study team. (Lines 164-169)

**2. What about genetic metabolic diseases? Were they investigated/excluded?**

**Response**

Thank you for this observation. We confirm that children with suspected or confirmed genetic or metabolic liver diseases were excluded from the study through medical history review and prior diagnostic testing. (Lines 174-176).

**3. Aim of the study: please revise the sentences at lines 139-144. They sound redundant, although the first sentence does not explain the kind of population “… in a the pediatric population”**

**Response**

We agree and have revised the aim section for clarity and precision. (lines 137-139)

**4. MASLD STAGES: how were they assessed? It seems that they refer to fibrosis stages. If so, please change the definition of MASLD stages into fibrosis stages (and consequently, modify X axis legend in figure 2), otherwise provide ref for such classification.**

**Response**

Yes, the “MASLD stages” referred to in the manuscript correspond to **fibrosis stages** as determined by transient elastography (FibroScan®). The terminology was corrected throughout the manuscript and the X-axis of Figure 2 was updated.

**5. SONOGRAPHIC FINDINGS: why did you use such classification? Are you sure it reflects the entity of liver steatosis and fibrosis? Please provide reference for such risk stratification.**

**Response**

We thank the reviewer for raising this point. Ultrasound classification was included in our study as one of the basic, routine, non-invasive tools for initial hepatic assessment. It is widely used for screening liver steatosis and fibrosis due to its accessibility, safety, low cost, and non-invasiveness. In pediatric MASLD, ultrasound serves as a first-line imaging modality to identify features such as hepatic echogenicity and hepatomegaly.

However, we would like to emphasize that ultrasound findings were **not the sole or determining measure** for staging liver fibrosis in this study. Fibrosis stages were assessed using **transient elastography (FibroScan®)**, which offers a more objective and quantifiable evaluation of liver stiffness. Ultrasound was used primarily to describe hepatic morphology and support the overall clinical assessment.

This was clarified in **lines 246 and 247**. Also, references were added

**6. TABLE 2: how correlations with MASLD stages and Ultrasonographic Category were performed? Pearson is for continuous variables.**

**Response**

We thank the reviewer. Pearson’s correlation was applied only to numeric variables. Consequently, MASLD stages and ultrasonographic categories—being categorical in nature—were not included in the Pearson correlation analysis, which is why “NA” appears in Table 2 for these variables. Instead, their associations with serum L‑FABP were assessed using analysis of variance (ANOVA) on top of regression modeling.

**7. A unique figure in the manuscript with different ROC curves for other NITS could be useful instead of several supplementary figures could be useful.**

**Response**

We thank the reviewer for this helpful suggestion. We have addressed it by consolidating the ROC curves of the non‑invasive tests (NITs) into one figure.

**8. liver biopsy were not performed, please mention it in the Limitation study section**

**Response**

Thanks for the comment. It was added as a limitation. (Line 456-457)

**MINOR REVISIONS**

- **REF 26: explications for Youden Index should be provided in methods section, not results**

**Response**

The reference was added in the method section (line 224)

- **Please provide ref for Chen, Chen, et al. (line 319)**

**Response**

It is Reference 34 (Chen Z-w, Chen L-y, Dai H-l, Chen J-h, Fang L-z. Relationship between alanine aminotransferase levels and metabolic syndrome in nonalcoholic fatty liver disease. J Zhejiang Univ Sci B. 2008;9:616-22.) in line 325

- Please provide reference for sentences at lines 129-130

**Response**

Added after that sentence instead of at the end of the paragraph (Reference 20)

- Sintax mistakes: lines 123-125; line 138; line 289 “being .. diagnosed MASLD”; along several lines there are unexplicable brackets (e.g. (MASLD) in the abstract, (F0-F1) at line 306)

**Response**

We thank the reviewer for their careful attention to the language and formatting. The identified issues have been thoroughly reviewed and corrected as follows:

- **Lines 123–125:**
  The sentence describing L-FABP has been revised for grammatical accuracy and clarity. The corrected version now reads:

*“Liver-type fatty acid-binding protein (L-FABP) is an antioxidant protein endogenously synthesized in the liver and the proximal tubular epithelial cells of the kidney, but not in skeletal or cardiac muscle.”*

- **Line 138:**
  A missing article was corrected. The phrase *“in paediatric population”* was revised to:

*“in the pediatric population”*

- **Line 289:**
  The phrasing *“being MASLD”* was revised to the grammatically correct form:

*“being diagnosed with MASLD”*

- **Unexplained Brackets:**
  All instances of unnecessary or unexplained parentheses—such as around “(MASLD)” in the abstract—have been reviewed and removed unless part of a formal abbreviation introduction. Technical bracketed terms like *(F0–F1)* were retained only when used appropriately to denote fibrosis staging.

These amendments have been reflected in the revised manuscript.
